# Polymorphisms in pattern recognition receptors and their relationship to infectious disease susceptibility in pigs

**DOI:** 10.1186/1753-6561-5-S4-S27

**Published:** 2011-06-03

**Authors:** Hirohide Uenishi, Hiroki Shinkai, Takeya Morozumi, Yoshihiro Muneta, Kosuke Jozaki, Chihiro Kojima-Shibata, Eisaku Suzuki

**Affiliations:** 1 National Institute of Agrobiological Sciences, Tsukuba, Ibaraki 305-8602, Japan; 2 Institute of Society for Techno-innovation of Agriculture, Forestry and Fisheries, Tsukuba, Ibaraki 305-0854, Japan; 3 National Institute of Animal Health, Tsukuba, Ibaraki 305-0856, Japan; 4 United Graduate School of Agricultural Sciences, Kagoshima University, Kagoshima 890-0065, Japan; 5 Miyagi Livestock Experimental Station, Miyagi Prefecture, Osaki, Miyagi 989-6445, Japan

## Abstract

**Background:**

Pattern recognition receptors (PRRs), including Toll-like receptors (TLRs), are censoring receptors for molecules derived from bacteria, viruses, and fungi. The PRR system is a prerequisite for proper responses to pathogens, for example by cytokine production, resulting in pathogen eradication. Many cases of polymorphisms in PRR genes affecting the immune response and disease susceptibility are known in humans and mice.

**Methods:**

We surveyed polymorphisms in pig genes encoding PRRs and investigated the relationship between some of the detected polymorphisms and molecular function or disease onset.

**Results:**

Nonsynonymous polymorphisms abounded in pig TLR genes, particularly in the region corresponding to the ectodomains of TLRs expressed on the cell surface. Intracellular TLRs such as TLR3, TLR7, and TLR8, and other intracellular PRRs, such as the peptidoglycan receptor NOD2 and viral RNA receptors RIG-I and MDA5, also possessed nonsynonymous polymorphisms. Several of the polymorphisms influenced molecular functions such as ligand recognition. Polymorphisms in the PRR genes may be related to disease susceptibility in pigs: pigs with a particular allele of *TLR2* showed an increased tendency to contract pneumonia.

**Conclusions:**

We propose the possibility of pig breeding aimed at disease resistance by the selection of PRR gene alleles that affect pathogen recognition.

## Background

As part of the innate immune system, both vertebrates and invertebrates possess pattern recognition receptors (PRRs), which respond to various kinds of molecules that characterize pathogens. Normally, PRR responses are quick, and their signals induce the production of proinflammatory cytokines and interferons, which are required for the appropriate exertion of other responses, including acquired immunity [1]. Vertebrates have fewer PRRs than invertebrates, but more than 30 genes encoding PRRs have been identified in each mammalian genome. In humans and mice, many studies have shown that some of the polymorphisms in PRR genes influence the immune response and disease susceptibility [2]. This suggests correlations between the immune response and PRR polymorphisms in pigs [3]. It is possible that these relationships could be used for breeding aimed at disease resistance. Here, we summarize the results of our searches for polymorphisms in PRRs and the influence of these polymorphisms on ligand recognition ability. The results of our preliminary study of the relationship between *TLR2* polymorphism and the onset of pneumonia imply that PRR polymorphisms also affect disease resistance in pigs.

## Methods

We used Landrace, Large White, Duroc, Berkshire, Hampshire, Middle Yorkshire, Clawn miniature, Potbelly, Meishan, and Jinhua breed pigs and Japanese Wild boars for polymorphisms detection [4-7]. Pig samples for exploration of polymorphisms were collected as ear notches, semen, peripheral blood, or muscle tissues, and genomic DNA was extracted by a standard method. cDNA was synthesized from RNA derived from peripheral blood. Polymorphisms were searched for by direct sequencing of PCR products covering the coding sequences generated with DNA and gene-specific primers. Targeted single nucleotide polymorphisms (SNPs) in additional samples were explored by mass spectrometry with a Sequenom MassARRAY Compact. To investigate the correlation between pneumonia and *TLR2* genotype, we fed 626 Landrace pigs on a *Mycoplasma hyopneumoniae* (Mhp)-positive farm until they weighed 105 to 115 kg. The pigs were then sacrificed and inspected for hepatization of the lung lobes as an indicator of pneumonia. The experiment was conducted in accordance with the animal experiment regulations of Miyagi Livestock Experiment Station.

## Results and discussion

### Distribution of polymorphisms in pig PRRs

We searched for polymorphisms in the coding sequences (CDSs) of TLRs (*TLR1*, *2*, *3*, *4*, *5*, *6*, *7*, and *8*), RIG-I-like helicases (*DDX58* encoding RIG-I and *IFIH1* encoding MDA5), and a NOD-like receptor (*NOD2*). We found many polymorphisms in the CDSs, particularly in the regions encoding domains required for ligand recognition [4-7]. Many SNPs changing the encoding of amino acids (nonsynonymous SNPs) were included in the ligand-recognizing regions. Notably, abundant nonsynonymous polymorphisms were observed in the ectodomains of TLRs expressed on cell surfaces (Table 1). In contrast, there were few nonsynonymous SNPs in the regions encoding domains related to signal transduction to the downstream pathways. (Table 1). The occurrence of polymorphisms in the domain in charge of ligand recognition may enable pig populations to cope with a broad variety of pathogens, whereas the occurrence of polymorphisms in the domain required for signal transduction has been strongly suppressed, possibly because of their harmful effects on the innate immunity of the hosts.

**Table 1:** Distribution of polymorphisms in CDSs of porcine PRRs

Gene	All			Region for ligand recognition			Region for signal transduction		
	
	All	NonSyn	Syn	All	NonSyn	Syn	All	NonSyn	Syn
*TLR1*	45	21	24	34	17	17	5	1	4
*TLR2*	23	11	12	17	10	7	3	1	2
*TLR3*	15	6	9	9	3	6	2	0	2
*TLR4*	13	7	6	10	7	3	1	0	1
*TLR5*	35	13	22	21	11	10	12	1	11
*TLR6*	20	11	9	14	10	4	2	0	2
*TLR7*	15	3	12	12	3	9	2	0	2
*TLR8*	22	9	13	20	7	13	0	0	0
*NOD2*	44*	14*	30	17	7	10	6	1	5
*DDX58* (RIG-I)	17	6	11	8	2	6	3	1	2
*IFIH1* (MDA5)	3	3	0	1	1	0	0	0	0

SNPs derived from 11 breeds, including Japanese wild boar (except for *NOD2*, *DDX58*, and *IFIH1*; six breeds of pig were used) were examined for polymorphisms. SNPs observed in the entire CDSs (All), in the ligand recognition region, and in the signal transduction region are indicated. Numbers of polymorphisms of *NOD2* include an insertion/deletion polymorphism located in the signal leader sequence (indicated by asterisks). NonSyn, nonsynonymous SNPs; Syn, synonymous SNPs.

### Functional influence of polymorphisms in pig PRRs on molecular recognition

Polymorphisms affecting the encoded amino acids in PRRs may influence molecular function—particularly ligand specificity. However, it is possible that the mutations remaining in the pig population have no effects, and that we can observe only those polymorphisms that are not harmful to molecular function. Therefore, we conducted reporter assays using expression vectors carrying the observed polymorphisms. Human cells (HEK293) into which expression vectors carrying some of the PRR polymorphisms had been introduced showed varying responses to a ligand. For example, the response of pig NOD2 with a 1949T→C mutation to muramyldipeptide was completely abolished, whereas that of pig NOD2 with 2197A→C showed an increased response to the ligand [4]. Furthermore, we have so far detected several nonsynonymous SNPs in *TLR2*, *TLR4*, and *TLR5* that affected their molecular function in ligand recognition (Shinkai et al., unpublished data).

### Possible correlation between PRR polymorphisms and onset of infectious diseases

The influence of the polymorphisms on ligand recognition ability suggested a relationship between disease susceptibility and PRR genotype. We surveyed the incidence of pneumonia caused mainly by *Mycoplasma hyopneumoniae* in a Landrace population and investigated genotypes of TLR2, which is responsible for recognition of Gram-positive bacteria. Among 626 pigs, 37 had a G nucleotide instead of a C at position 406 in the CDS, causing an amino acid alteration from proline to alanine. Seventy-six percent (28 animals) of pigs with the G allele showed histopathological signs of pneumonia, whereas 57% (331 animals) of those without the G allele had pneumonia (Table 2). The former had a significantly greater incidence of pneumonia than the latter (odds ratio = 2.30; 95% interval: 1.07–4.97; *P* = 0.038). This suggests a relationship between PRR genotype and disease susceptibility, although we have to examine the susceptibility of animals with the homozygous G alleles to pneumonia. Further investigation with other pig populations may elucidate correlations between other SNPs in PRRs and disease susceptibility.

**Table 2:** Relationship between *TLR2* genotype and occurrence of pneumonia

Genotype	Cases	Controls
	
	*n*	*n*
C/C	331	245
C/G	28	9

Pigs of a Landrace population were used to investigate the occurrence of pneumonia caused mainly by *Mycoplasma hyopneumoniae*. Genotypes were demonstrated by changes in the nucleotide at position 406 in the CDS.

## Conclusions

Many polymorphisms occur in the genes encoding PRRs in pigs. Some of the polymorphisms have an influence on ligand recognition ability. Polymorphisms in PRRs may be correlated with disease resistance in pigs.

## Abbreviations

PRR: pattern recognition receptors; TLR: Toll-like receptors; CDS: coding sequence.

## Competing interests

The authors declare that they have no competing interests in this study.

## Authors’ contributions

HU, HS, TM, and YM designed the study. HU, HS, TM, KJ, and CKS detected the polymorphisms. Reporter assays were performed by HS, TM, and KJ. HS, YM, CKS, and ES performed the pneumonia study. HU wrote the manuscript.
